# Integrative single-cell and exosomal multi-omics uncovers SCNN1A and EFNA1 as non-invasive biomarkers and drivers of ovarian cancer metastasis

**DOI:** 10.3389/fimmu.2025.1630794

**Published:** 2025-07-25

**Authors:** Liping Tang, Dong Pang, Chengbang Wang, Jiali Lin, Shaohua Chen, Jiangchun Wu, Junqi Cui

**Affiliations:** ^1^ Department of Rehabilitation Medicine, Pudong New District Gongli Hospital, Shanghai, China; ^2^ Department of Obstetrics and Gynecology, The People’s Hospital of Guangxi Zhuang Autonomous Region, Guangxi Academy of Medical Sciences, Nanning, China; ^3^ Department of Urology, Shanghai Ninth People’s Hospital, Shanghai Jiao Tong University School of Medicine, Shanghai, China; ^4^ Department of Pathology, Shanghai Ninth People’s Hospital, Shanghai Jiao Tong University School of Medicine, Shanghai, China; ^5^ Department of Urology, Guangxi Medical University Cancer Hospital, Nanning, Guangxi, China; ^6^ Department of Gynecologic Oncology, Fudan University Shanghai Cancer Center, Shanghai Medical College, Fudan University, Shanghai, China

**Keywords:** ovarian cancer, single-cell RNA sequencing, exosome, biomarker, metastasis

## Abstract

**Background:**

Ovarian cancer (OV) is the deadliest gynecologic malignancy owing to its late diagnosis and high metastatic propensity. Current biomarkers lack sufficient sensitivity and specificity for the detection of early-stage cancer. To address this gap, we integrated single‐cell transcriptomic profiling of tumor tissues with analysis of circulating exosomal RNA, aiming to uncover candidate markers that reflect tumor heterogeneity and metastatic potential and that may serve as sensitive, non‐invasive diagnostics.

**Methods:**

We integrated single-cell RNA sequencing (scRNA-seq) data from primary tumors and metastatic lesions with bulk tissue transcriptomes and plasma-derived exosomal RNA sequencing (RNA-seq). Differentially expressed genes (DEGs) shared across tumor cells, metastatic subpopulations, and exosomes were identified through intersection analysis. Candidate genes were validated in clinical specimens using qPCR and immunohistochemistry. We then applied ten machine learning algorithm to exosomal transcriptomic data to evaluate diagnostic performance and identify the optimal classifier. Tumor cell differentiation states were evaluated using CytoTRACE, and intercellular communication was analyzed with CellChat.

**Results:**

Intersection analysis highlighted 52 overlapping DEGs, of which SCNN1A and EFNA1 emerged as the top prognostic indicators. Both genes were significantly upregulated in tumor tissues, metastatic foci, and plasma exosomes (*P* < 0.01). The exosome-based Adaboost model had an area under the curve of 0.955 in an independent test cohort. Single-cell subcluster analyses revealed high SCNN1A/EFNA1 expression correlated with stem-like differentiation states and enriched pathways associated with immune evasion and adhesion. CellChat analysis demonstrated that highly differentiated tumor cells extensively engaged with fibroblasts and endothelial cells, implying their role in niche formation.

**Conclusions:**

By coupling single-cell, bulk tissue, and exosomal transcriptomics, we elucidated the key molecular drivers of OV metastasis and established SCNN1A and EFNA1 as promising non-invasive biomarkers. This multi-omics framework provides an effective strategy for early detection and a better understanding of metastatic progression in OV.

## 1 Introduction

Ovarian cancer (OV) is the most lethal cancer of the female reproductive system, accounting for 3.4% of new female cancer cases in 2020 and ranking as the eighth leading cause of cancer-related deaths in women worldwide (1). Its poor prognosis is largely due to aggressive metastatic behavior and a pronounced ability to evade immune surveillance. The 5-year survival rate for patients with advanced-stage disease remains below 30%, although early diagnosis can improve survival rates to over 90% (2, 3). This is an ideal strategy for improving the survival rate of patients with OV. However, due to the lack of specific early symptoms, more than 70% of patients are diagnosed at FIGO stage III/IV (4). Cancer antigen 125 (CA125) is commonly used for OV screening; however, its diagnostic efficacy depends on the disease risk and stage. CA125 levels are elevated in only 50% of stage I epithelial OVs (5), and their specificity is further compromised by benign diseases, such as endometriosis, uterine fibroids, and menstrual cycle abnormalities (6). Transvaginal ultrasonography, another screening method, does not reduce mortality in asymptomatic women and may increase the risk of damage (7, 8). Accordingly, uncovering the mechanisms of OV progression and developing highly sensitive early diagnostic biomarkers are important for precision medicine and therapeutic decisions.

Recently, advances in high-throughput sequencing technologies and multi-omics analyses have enabled researchers to analyze the molecular etiology of OV systematically (9). Single-cell transcriptome sequencing (scRNA-seq) has enabled RNA profiling at the cellular level, serving as a high-precision technique for unraveling interaction networks between epithelial, immune, and stromal cells in the tumor microenvironment (TME) (10). However, studies solely focused on tumor tissues may overlook potential biomarker signals in the peripheral circulation, which involve dynamic interactions between the TME and the host system.

Blood-derived extracellular vesicles (EVs) offer a promising avenue for investigating the mechanisms of cellular interactions in OV metastasis and immune evasion and identifying diagnostic biomarkers. Exosomes are cell-derived vesicles ranging from 30 to 150 nm in diameter, initially considered membrane-derived fragments involved in cellular waste disposal (11). However, extensive research has revealed that they are critical mediators of intercellular communication. These vesicles are actively secreted by OV cells and contain diverse molecular cargo, including RNA, DNA, proteins, and metabolites (12). They actively participate in cellular communication in the TME and play an important role in immune evasion (13, 14) and metastatic cell migration (15). Emerging evidence highlights the clinical relevance of exosomal mechanisms. Su et al. identified exosomal miR-375 and miR-1307 as complementary biomarkers to CA125, with high sensitivity and specificity in distinguishing early-stage OV patients from healthy controls. These miRNAs improve diagnostic accuracy when combined with the protein biomarkers CA125 and HE4 (16). However, studies focusing on single exosomal components still have limitations, particularly in linking their cellular origins and functional mechanisms.

This study addressed these limitations by integrating scRNA-seq with bulk RNA sequencing (RNA-seq) and blood-derived exosome transcriptome data analyses. We analyzed scRNA-seq datasets from primary tumors and metastatic lesions to identify a group of TME-related differentially expressed genes (DEGs). We investigated the expression patterns of these DEGs and their relevance to OV pathogenesis using bulk RNA data from The Cancer Genome Atlas (TCGA) and Genotype-Tissue Expression (GTEx) databases, combined with scRNA-seq profiles. Furthermore, we used blood-derived exosomal RNA sequencing to determine the cellular origin of the mRNAs contained in EVs. We validated the expression of SCNN1A and EFNA1 in primary tumors, EVs, and metastatic lesions. We validated the expression of selected candidate genes in primary tumors, EVs, and metastatic lesions. Based on their expression profiles, we developed a machine learning-based diagnostic model that achieved high accuracy for early-stage OV detection. To further elucidate their biological significance, we investigated their association with tumor invasiveness and immune evasion, thereby uncovering potential mechanisms driving OV progression.

## 2 Materials and methods

### 2.1 Data acquisition

This study combined nine independent datasets obtained from publicly available repositories. Particularly, six scRNA-seq datasets from the Gene Expression Omnibus (GEO) database: GSE154600 (OV, n = 5), GSE158937 (OV, n = 3) (17), GSE173682 (OV, n = 4) (18), GSE184880 (OV, n = 7, normal ovarian tissue, n = 5) (19), GSE158722 (OV pleural effusion, n = 39) (20), and GSE186344 (OV brain metastasis, n = 2) (21). Furthermore, data from TCGA for OV and the GTEx database for normal ovarian tissue were incorporated, resulting in 469 samples (381 tumor and 88 adjacent non-tumor tissues) with corresponding clinical annotations. Bulk RNA-seq data of blood-derived exosomes from patients with OV and healthy controls were obtained from the exoRbase database (http://www.exorbase.org/), comprising 148 samples (30 tumor and 118 healthy controls).

### 2.2 scRNA-seq data processing

scRNA-seq data were analyzed using the *Seurat* package (version 5.1.0) (22). Low-quality cells were excluded if they contained < 200 or > 11,000 detected genes or their mitochondrial RNA content exceeded 25% (19, 23). Data normalization and dimensionality reduction were performed using the “SCTransform,’’ “RunPCA,’’ and “RunUMAP” functions. Cellular identities were determined using the *scHCL* (version 0.1.1; https://github.com/ggjlab/scHCL) and *SingleR* (version 1.10.0; https://github.com/dviraran/SingleR) packages. Subsequently, the “FindAllMarkers” function in *Seurat* was used to identify marker genes specific to each cell subpopulation, and cell types were assigned based on previously published markers. For tumor cell identification, the unique molecular identifier count matrix served as the input for inferring chromosomal copy number alteration profiles using the *inferCNV* R package (version 1.3.3; https://github.com/broadinstitute/inferCNV).

### 2.3 Differential gene expression analysis

We performed differential expression analyses as follows. For scRNA-seq data, we applied the “FindMarkers” function in the *Seurat* package, with significance defined by *P* < 0.05 and |log_2_FC| > 0.25. For TCGA and GTEx OV cohorts, DEGs were identified using the *DESeq2* (version 1.36.0), *limma* (version 3.52.4), and *edgeR* (version 3.38.4) packages, we selected the limma-voom results for all downstream analyses and visualizations due to its robustness with large, heterogeneous datasets, with stricter thresholds of *P* < 0.05 and |log_2_FC| > 1, and we selected limma-voom for all downstream analyses and visualizations because of its computational efficiency and robustness with large, heterogeneous datasets. Similarly, DEGs in the bulk RNA-seq data of blood-derived exosomes were determined using *P* < 0.05 and |log_2_FC| > 0.5, with subsequent analysis based on limma-voom. The intersection of DEGs across these datasets was then visualized using the *UpSetR* package (version 1.4.0).

### 2.4 Functional analysis

The *clusterProfiler* package (version 4.4.4) was used to perform functional enrichment analysis of DEGs in Gene Ontology (GO) and Kyoto Encyclopedia of Genes and Genomes (KEGG) databases. Enriched terms were selected using a significance cutoff of *P* < 0.05, and the results were visualized using *ggplot2* (version 3.5.1).

### 2.5 Clinical correlation and survival analysis

We used GEPIA2.0 (http://gepia2.cancer-pku.cn/#index, accessed on July 7, 2024), a data visualization platform for the TCGA database, to assess the effects of candidate biomarker genes on overall survival in OV. Kaplan-Meier survival curves were generated, and the correlations between key genes and clinical indicators were examined.

### 2.6 Machine learning analysis

A stratified random sampling method was used to divide the exoRBase OV cohort into training and testing groups in an 8:2 ratio. This process used the “initial_split” function of the *rsample* package (version 1.2.1) to conduct stratified sampling of the tumor and healthy groups. Genes were screened using the “glm” function and the “cv.glmnet” function of the *glmnet* package (version 4.1-2). Ten widely used machine learning algorithms were then trained—AdaBoost, gradient boosting machine (Gbm), elastic net (Glmnet), k-nearest neighbors (kknn), LogitBoost, multilayer perceptron (MLP), naïve Bayes (NB), regularized logistic regression (RegLogistic), random forest (RF), and support vector machine with radial basis kernel (svmRadialWeights). Each model underwent tenfold cross-validation, and the entire resampling process was repeated three times to reduce variability. Model performance was evaluated by AUC, accuracy, recall, precision, F1 score, and Cohen’s kappa, calculated using the “twoClassSummary”, “postResample”, and “confusionMatrix” functions from the *caret* package (v7.0-1). Radar plots comparing these metrics were generated with *ggradar* (v0.2) and *ggplot2*.

### 2.7 Differentiation states prediction

Differentiation states or stemness status in the scRNA-seq data were estimated using the R package *CytoTRACE* (version 0.3.3) (24). This robust computational framework ranks single cells based on gene counts, indicating transcriptome diversity and relative developmental potential. CytoTRACE scores ranged from 0 to 1, with higher scores indicating greater stemness or reduced differentiation and vice versa.

### 2.8 Cell-cell interaction network analysis

*CellChat* (version 2.1.2) (25), an R package designed explicitly for cell-cell communication analysis, was used to identify ligand-receptor interactions in scRNA-seq data. This package provides a comprehensive toolkit for analyzing cellular communication networks. By evaluating ligand expression in one cell population alongside corresponding receptor expression in other cell types, we delineated putative signaling pathways, thereby deepening our understanding of intercellular crosstalk in complex biological systems.

### 2.9 Sample acquisition

All clinical samples were obtained with the approval of the Institutional Review Board of the Shanghai Cancer Center (Approval No: 050432-4-2307E). Written informed consent was obtained from all patients in compliance with the ethical principles of the Declaration of Helsinki. OV tissue samples, including tumor and adjacent non-cancerous tissues, were collected from patients who underwent surgery at the Shanghai Cancer Center. Immediately after excision, the tissue samples were fixed in 10% formalin and embedded in paraffin (FFPE) for histological analysis. FFPE tissues were subsequently processed for IHC analysis to confirm the expression of key biomarkers, including SCNN1A and EFNA1.

Peripheral blood samples were collected from patients with OV at the Shanghai Cancer Center. Plasma was separated by centrifugation at 1,500 × *g* for 10 min, followed by a second centrifugation at 12,000 × *g* for 15 min to remove residual cellular debris. Exosomes were then isolated from plasma for downstream analyses, including RNA extraction and expression profiling.

### 2.10 Immunohistochemistry and quantitative analysis

Tissue samples from four cases of primary OV and their paired adjacent non-cancerous tissues, as well as four metastatic OV lesions (bladder, colon, liver, and lung metastases), were collected for analysis. All samples were fixed, dehydrated, and embedded for subsequent sectioning. The sections were deparaffinized with xylene and rehydrated with graded ethanol before antigen retrieval in citrate buffer (pH 6.0) under high pressure. Endogenous peroxidase activity was blocked with hydrogen peroxide. Primary antibodies targeting EFNA1 (1:200 dilution) and SCNN1A (1:300 dilution) were used, and the sections were incubated overnight at 4°C. After three washes with phosphate-buffered saline, HRP-conjugated secondary antibodies were added, and the sections were incubated at room temperature for 1 h. DAB was used for color development, followed by counterstaining with hematoxylin, dehydration, and mounting. Positive staining areas were semi-quantitatively analyzed using ImageJ software to compare the protein expression levels across the groups.

### 2.11 Extracellular vesicle isolation

Exosomes were isolated from the plasma of six patients with OV and six healthy controls using the sEV 500 Ultrapure Columns kit. Plasma samples were centrifuged at 3,000 *× g* for 10 min at 4°C to remove cell debris, and the supernatant was carefully transferred to a new tube. The supernatant was centrifuged again at 10,000 *× g* for 30 min at 4°C, and the resulting supernatant was collected for further processing. Before loading the samples, the purification columns were cleaned and equilibrated according to the manufacturer’s instructions. The processed supernatant (1–2 mL) was added to the column filter plate. Once the sample had fully entered the filter plate and no liquid was visibly flowing out from the bottom, an elution buffer (4.5 mL - sample volume) was immediately added to ensure a total flow-through of 4.5 mL with no EVs. Next, a fresh EP tube was placed under the column, and 0.9 mL of the elution buffer was added. The collected 0.9 mL exosome-enriched eluate was immediately transferred to a -80°C freezer for long-term storage.

The isolated exosomes were characterized using nanoparticle tracking analysis to assess the particle size distribution (**Supplementary Table 1**). Validation experiments included transmission electron microscopy to observe the characteristic round vesicle structures of exosomes (**Supplementary Figure 1A**). For Western blotting (WB) (**Supplementary Figure 1B**), exosomes were lysed in RIPA buffer containing protease inhibitor and centrifuged at 15,000×g for 20 min at 4°C. Protein concentration of the supernatant was measured by BCA assay. Samples were mixed with 5× loading buffer, denatured at 99°C for 15 min, and 30 µg of protein was separated on 10% SDS–PAGE gels (120 V, 2 h). Proteins were transferred to PVDF membranes at 300 mA for 2 h, then blocked in 5% BSA for 2 h at room temperature. Membranes were incubated overnight at 4°C with primary antibodies against CD63, CD81, and TSG101, washed, and then incubated with HRP-conjugated secondary antibodies for 1 h at room temperature. Blots were developed with ECL substrate and imaged.

### 2.12 RNA extraction

Total RNA was extracted from the exosome samples using the QIAGEN miRNeasy Micro Kit (Cat. No. 217084) according to the manufacturer’s instructions. Briefly, 500 µL of exosome samples were lysed in 1 mL of QIAzol Lysis Reagent and incubated at room temperature for 5 min. After adding 200 µL of chloroform, the mixture was shaken, incubated for 2–3 min, and centrifuged at 12,000 × *g* for 15 min at 4°C. The aqueous phase was carefully transferred to a new tube, mixed with an equal volume of absolute ethanol, and loaded onto an RNeasy MinElute spin column. The column was sequentially washed with Buffer RWT and RPE, followed by final centrifugation to remove the residual buffer. RNA was eluted with 14 µL of preheated RNase-free water and collected in a 1.5 mL tube. Purified RNA was stored appropriately for downstream analysis.

### 2.13 RNA reverse transcription

cDNA was synthesized using the Takara RR047A kit in a two-step reverse transcription process that included gDNA digestion and cDNA synthesis. In the first step, 10 µL RNA was combined with 2.0 µL of 5× gDNA Eraser Buffer and 1.0 µL of gDNA Eraser in a total reaction volume of 13 µL. The mixture was gently mixed and incubated in a PCR instrument at 42°C for 2 min, after which it was maintained at 4°C. In the subsequent reverse transcription reaction, 13.0 µL of the gDNA digestion product was mixed with 1.0 µL PrimeScript RT Enzyme Mix I, 1.0 µL RT Primer Mix, 4.0 µL of 5× PrimeScript Buffer 2 (for Real Time), and 1.0 µL RNase-free dH_2_O to yield a total volume of 20 µL. After gentle mixing, the reaction was incubated at 37°C for 15 min, followed by incubation at 85°C for 5 s, and then held at 4°C.

### 2.14 Target primer design, synthesis, and validation

The target gene sequence was retrieved from the Ensembl database, and qPCR primers were designed using the online NCBI Primer Designing Tool (https://www.ncbi.nlm.nih.gov/tools/primer-blast/index.cgi?LINK_LOC=BlastHome). The melting temperature of each primer pair was determined. Using cDNA from PC3 prostate cancer cells as the template, PCR amplifications were performed with TB Green^®^ Premix Ex Taq™ II over 35 cycles at three different annealing temperatures (58, 59, and 60 °C). Subsequently, 5 µL of each PCR product was analyzed by agarose gel electrophoresis to select candidate primers with optimal amplification efficiency and determine the best annealing temperature. The dissociation curve for each candidate primer pair was verified using a dye-based method under optimal conditions.

Further melting temperature analysis allowed the establishment of three to four annealing temperature gradients. Diluted primers and PC3 cell cDNA were used for subsequent PCR amplifications with TB Green II under the following cycling conditions: initial denaturation at 95 °C for 30 s; 35 cycles of 95 °C for 5 s, annealing at 60 °C for 30 s, and extension at 72 °C for 15 s; final extension at 72 °C for 1 min; and hold at 4 °C. The amplification reaction mixture consisted of 2 µL cDNA, 5 µL of 2× TB Green, 0.4 µL each of forward and reverse primers (10 µM), and 2.2 µL ddH_2_O (primer details are provided in **Supplementary Table 2**). Statistical analysis of gene expression differences between groups was performed using the R language, with significance at *P* < 0.05.

## 3 Results

### 3.1 Profiling of ovarian tissue scRNA-seq, bulk RNA-seq, and blood-derived exosome RNA-seq

**Figure 1:**
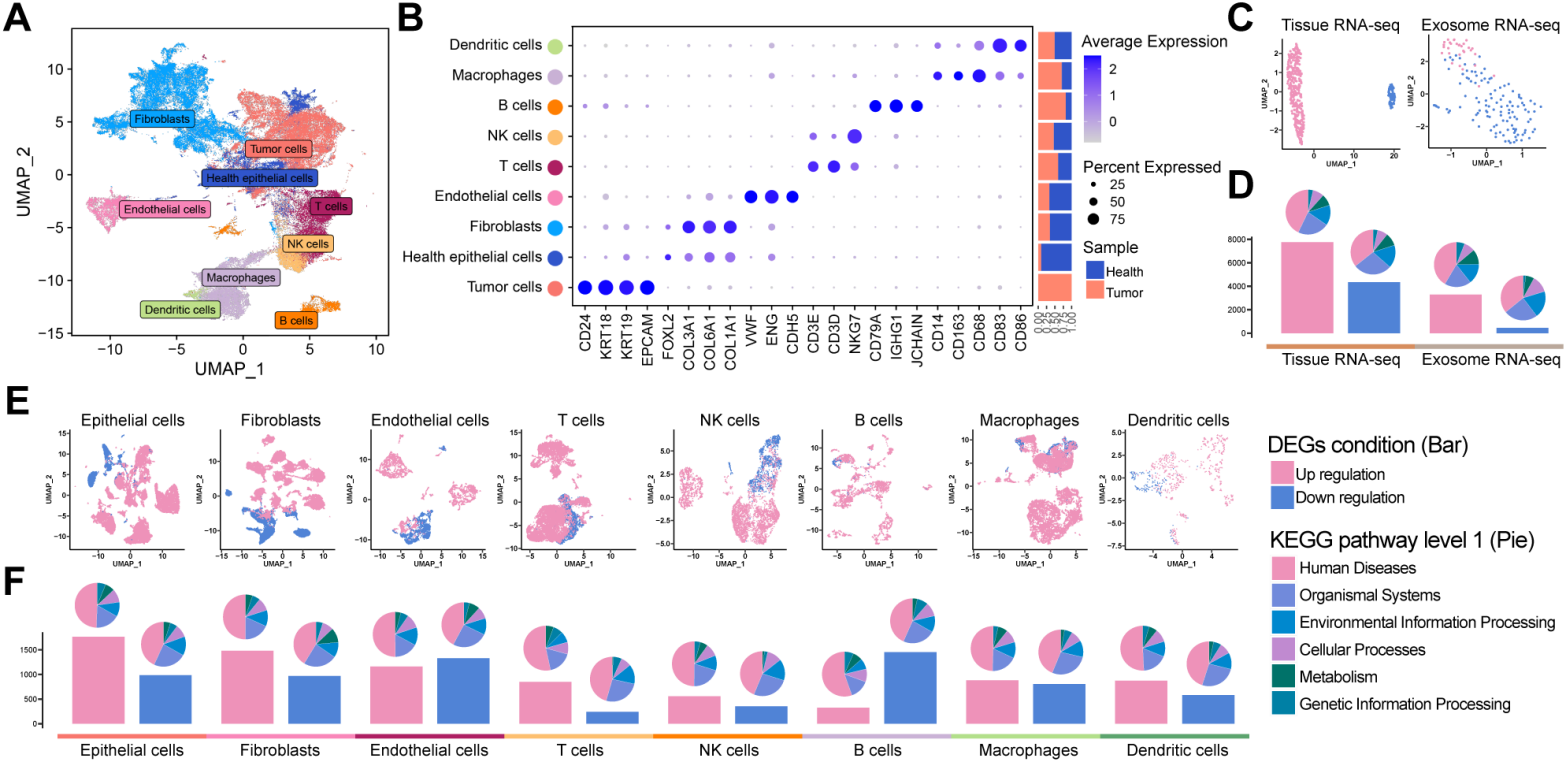
Tumor tissue atlas: **(A)** UMAP plot of OV tissue scRNA-seq. **(B)** Marker gene distribution of major cell types, with annotations for cell type, classic markers, and origin ratio. **(C)** UMAP for TCGA bulk RNA-seq, GTEx, and OV exosome bulk RNA-seq, differentiating tumor (pink) and control (blue) samples. **(D)** Bar plot of DEGs with tumor-upregulated (pink) and downregulated (blue) DEGs, along with enriched pathway proportions (KEGG). **(E, F)** Single-cell sub-cluster UMAPs and DEGs bar plots demonstrating differences between the tumor and control groups.

To comprehensively characterize the cellular and molecular landscape of OV, we integrated four scRNA-seq datasets from patients with OV and healthy controls. After rigorous quality control, 112,685 high-quality single cells were selected for downstream analysis. Dimensionality reduction using uniform manifold approximation and projection (UMAP) revealed distinct clusters corresponding to nine major cell types (**Figure 1**), including healthy epithelial cells, tumor cells, fibroblasts, endothelial cells, T cells, natural killer cells, B cells, macrophages, and dendritic cells, along with their specific marker genes (**Figure 1**). Notably, TME exhibited a higher proportion of immune cells than normal ovarian tissue.

We analyzed bulk RNA-seq datasets from TCGA and GTEx projects and blood-derived exosomal RNA-seq data from patients with OV and healthy controls to further elucidate the molecular differences between tumor and normal samples. UMAP revealed robust separation between tumor and healthy groups in both tissue-derived and exosomal transcriptomic datasets (**Figure 1**). Differential expression analysis consistently revealed a predominance of upregulated DEGs on both platforms, as illustrated by the bar plots (**Figure 1**, **Supplementary Tables 3**, **4**). Similarly, scRNA-seq data confirmed these trends: UMAP visualizations revealed distinct transcriptomic profiles between tumor and normal cells (**Figure 1**), and differential expression analysis within specific subpopulations indicated that epithelial cells harbored over 1,500 upregulated DEGs, with fibroblasts and endothelial cells also indicating significant increases, whereas B cells exhibited the highest number of downregulated DEGs (**Figure 1**). KEGG pathway enrichment analysis across all datasets highlighted significant associations with human disease-related pathways.

### 3.2 Cellular atlas of OV metastasis by scRNA-seq

**Figure 2:**
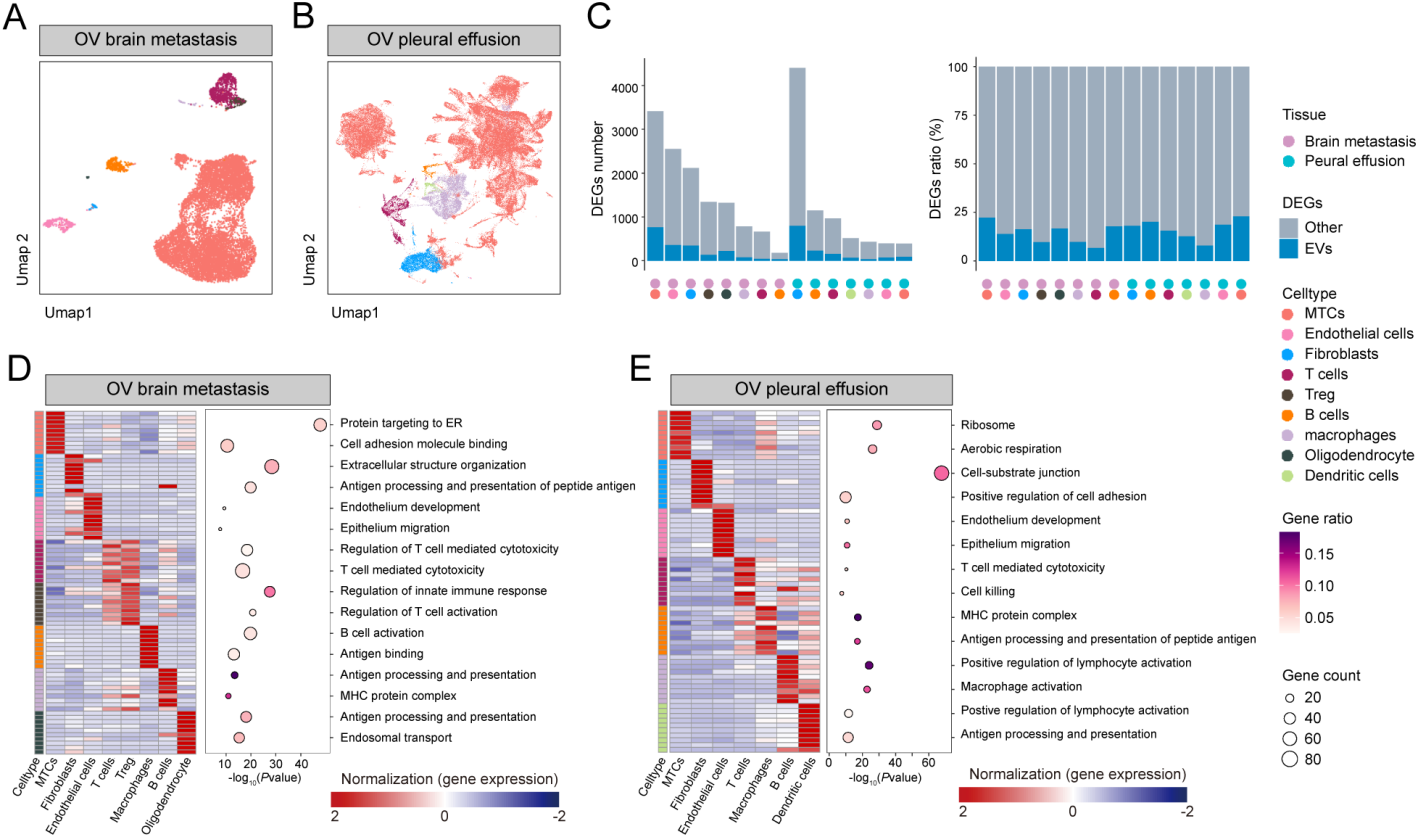
Metastatic tissue atlas: **(A, B)** UMAP of brain metastasis and pleural effusion scRNA-seq samples. **(C)** DEG count and proportion plot highlighting the intersection of OV exosome upregulated DEGs and DEGs of differential cell types. **(D, E)** Heatmap of high-expression genes and bubble plot of GO functional enrichment analysis.

Based on these findings, we investigated the metastatic landscape of OV by analyzing scRNA-seq data from two metastatic sites. Comprehensive transcriptomic atlases were prepared for both brain and pleural effusion-derived metastases, providing a continuum from primary tumor alterations to metastatic dissemination (**Figures 2A**). Eight distinct cell types were identified in brain metastases, including metastatic tumor cells (MTCs), endothelial cells, fibroblasts, oligodendrocytes, B cells, macrophages, T cells, and regulatory T cells. Conversely, pleural effusion-derived metastases comprised seven cell types: MTCs, endothelial cells, fibroblasts, oligodendrocytes, B cells, macrophages, and T cells. Notably, MTCs accounted for the highest proportion of cells in both datasets. Given their predominance and critical role in tumor dissemination, subsequent analyses focused primarily on MTCs.

For each cell type, we performed differential expression analysis against all other cells, then intersected the resulting DEGs with those from blood‐derived exosomal RNA-seq data to assess both the number and the proportion of cell‐specific DEGs represented in circulating exosomes (**Figure 2**, **Supplementary Table 5**). MTCs exhibited the most upregulated DEGs in brain metastasis samples and demonstrated the greatest overlap with exosome-associated upregulated DEGs (**Supplementary Table 6**). Conversely, in pleural effusion metastasis samples, fibroblasts had the highest number of DEGs, whereas MTCs had the fewest (**Supplementary Table 7**). Interestingly, exosome-associated upregulated DEGs were proportionally enriched in MTCs from pleural effusion samples, consistent with brain metastasis findings.

To investigate the biological pathways driving site-specific tumor behavior, we performed enrichment analyses on DEGs selectively upregulated in the main cell populations of each metastatic sample. In brain metastases (**Figure 2** and **Supplementary Table 8**), MTC DEGs were primarily enriched in pathways related to protein targeting to the endoplasmic reticulum and cell adhesion molecule binding, suggesting that cell-cell and cell-matrix adhesion processes may contribute to successful tumor colonization at solid metastatic sites. In pleural effusion metastases (**Figure 2** and **Supplementary Table 9**), MTC DEGs were enriched in ribosome biogenesis and aerobic respiration pathways, consistent with an increased energetic demand that could accompany the metastatic process.

These comprehensive analyses provide valuable insights into the cellular heterogeneity and molecular changes associated with OV metastases, establishing a rigorous framework for identifying potential diagnostic biomarkers and therapeutic targets.

### 3.3 Intersectional analysis, validation, and diagnostic potential of key genes

**Figure 3:**
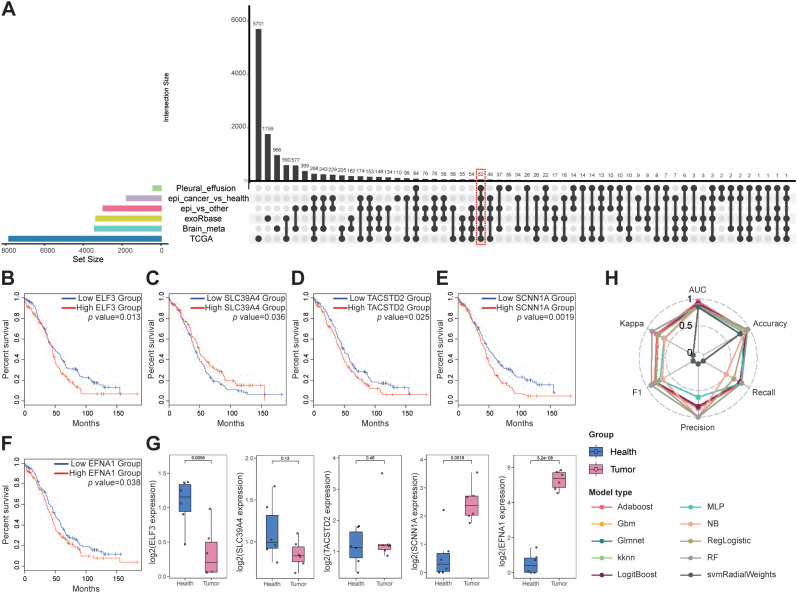
Biomarker discovery for OV metastasis: **(A)** UpSet plot for intersecting gene analysis. **(B, F)** Survival analysis of the key genes. **(G)** qPCR validation of key genes in the plasma exosomes. **(H)** Classification performance of ten machine-learning models, assessed by six metrics: AUC, accuracy, recall, precision, F1 score, and Cohen’s kappa.

Based on transcriptomic observations in OV, we sought to identify robust biomarkers through comprehensive differential expression analysis. By intersecting DEGs across multiple datasets, we pinpointed genes consistently associated with the tumor epithelium, blood-derived exosomes, and metastatic lesions. The UpSet plot (**Figure 3** and **Supplementary Table 10**) displays the intersection of six transcriptomic datasets: 1) Epi vs other: DEGs upregulated in OV epithelial cells versus all other tumor cell types; 2) Epi cancer vs health: DEGs upregulated in OV epithelial cells versus normal ovarian epithelial cells; 3) TCGA: DEGs upregulated in TCGA bulk RNA-seq OV samples versus controls; 4) Brain meta: DEGs upregulated in MTCs from brain metastases versus other brain cell types; 5) Pleural effusion: DEGs upregulated in MTCs from pleural effusion metastases versus other effusion cell types; and 6) exoRbase: DEGs upregulated in blood-derived exosomes from OV patients versus healthy controls. From these intersections, we identified 52 key genes.

Survival analysis and clinical stage correlations were then performed on these 52 genes, and five key genes were identified: ELF3, SLC39A4, TACSTD2, SCNN1A, and EFNA1, all of which were significantly associated with patient prognosis (**Figures 3B**, **Supplementary Table 11**). Notably, overexpression of ELF3, TACSTD2, SCNN1A, and EFNA1 was associated with poorer survival in patients with OV, whereas high expression of SLC39A4 was associated with better prognosis, although overexpression was typically observed in later-stage tumors (**Supplementary Figure 2**).

Plasma samples from six patients with OV and six healthy controls were analyzed to confirm these findings. Exosomes were isolated from these samples, and qPCR analysis confirmed that SCNN1A and EFNA1 expression was significantly upregulated in patients with OV compared to the controls (*P* < 0.01; **Figure 3**). We evaluated ten machine learning classifiers, including Adaboost, Gbm, Glmnet, kknn, LogitBoost, MLP, NB, RegLogistic, RF and svmRadialWeights, using the expression of these two genes as features. The exosome dataset was split into training (80%) and testing (20%) cohorts for each model. All models demonstrated strong diagnostic performance, with the Adaboost model achieving the highest AUC of 0.955 on the test set (**Figure 3**, **Supplementary Table 12**). These findings confirm SCNN1A and EFNA1 as promising biomarkers for non-invasive OV diagnosis, highlighting their critical roles in OV progression and metastasis and providing a strong foundation for future clinical applications.

**Figure 4:**
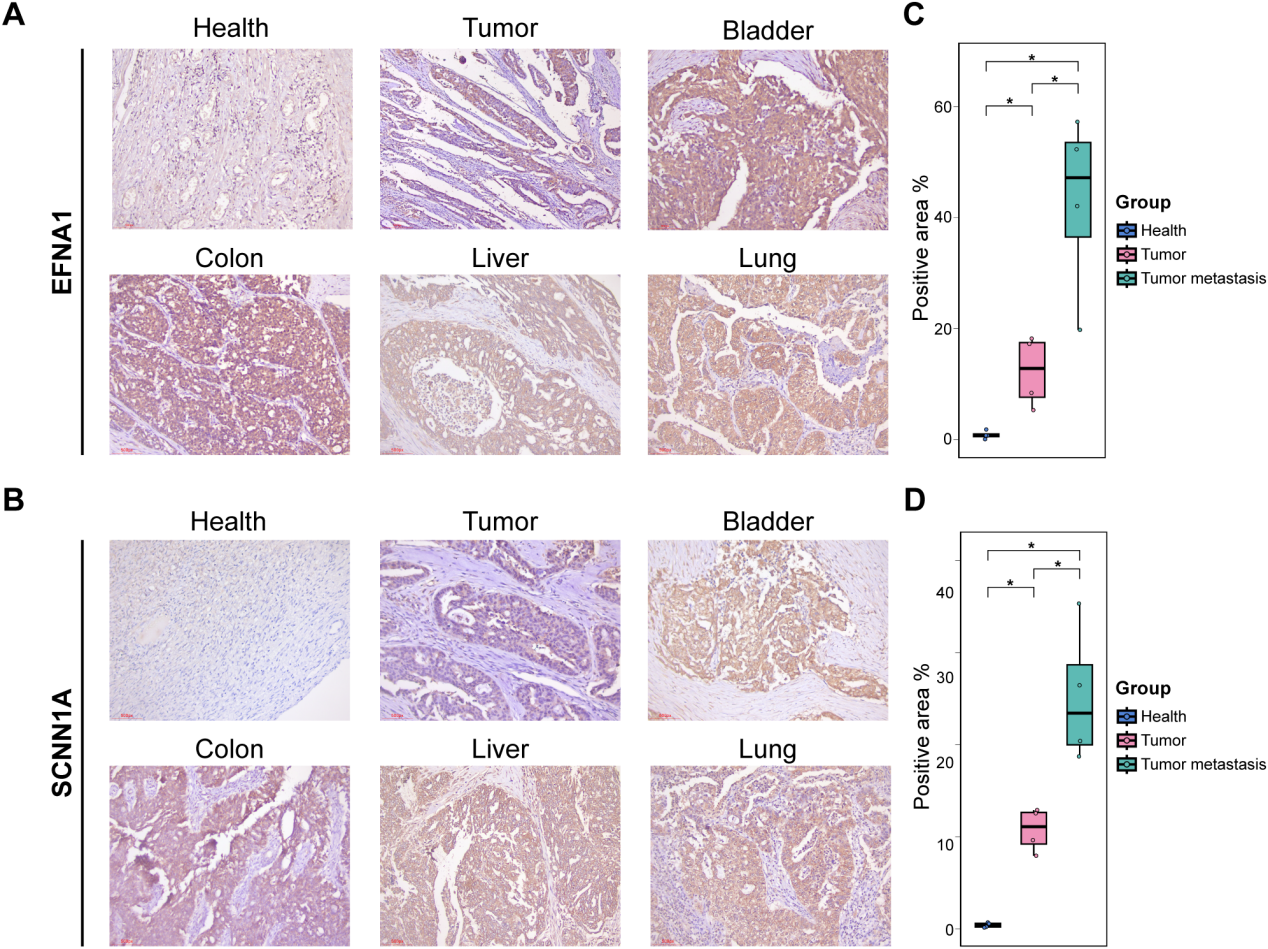
IHC validation: **(A, B)** IHC staining for EFNA1 and SCNNs in different tissue sections. **(C, D)** Quantification of positive staining regions. *P < 0.05.

Furthermore, immunohistochemistry (IHC) was performed on four pairs of primary OV tissues with matched adjacent non-tumor tissues and on OV metastatic samples from the bladder, colon, liver, and lung to corroborate the reliability of these biomarkers further. IHC analyses revealed significantly elevated protein expression of SCNN1A and EFNA1 in OV tissues compared to controls (**Figures 4A**), with metastatic lesions exhibiting significantly higher expression than primary tumors (*P* < 0.05; **Figures 4C**).

### 3.4 Tumor cell subcluster and differentiation analysis

**Figure 5:**
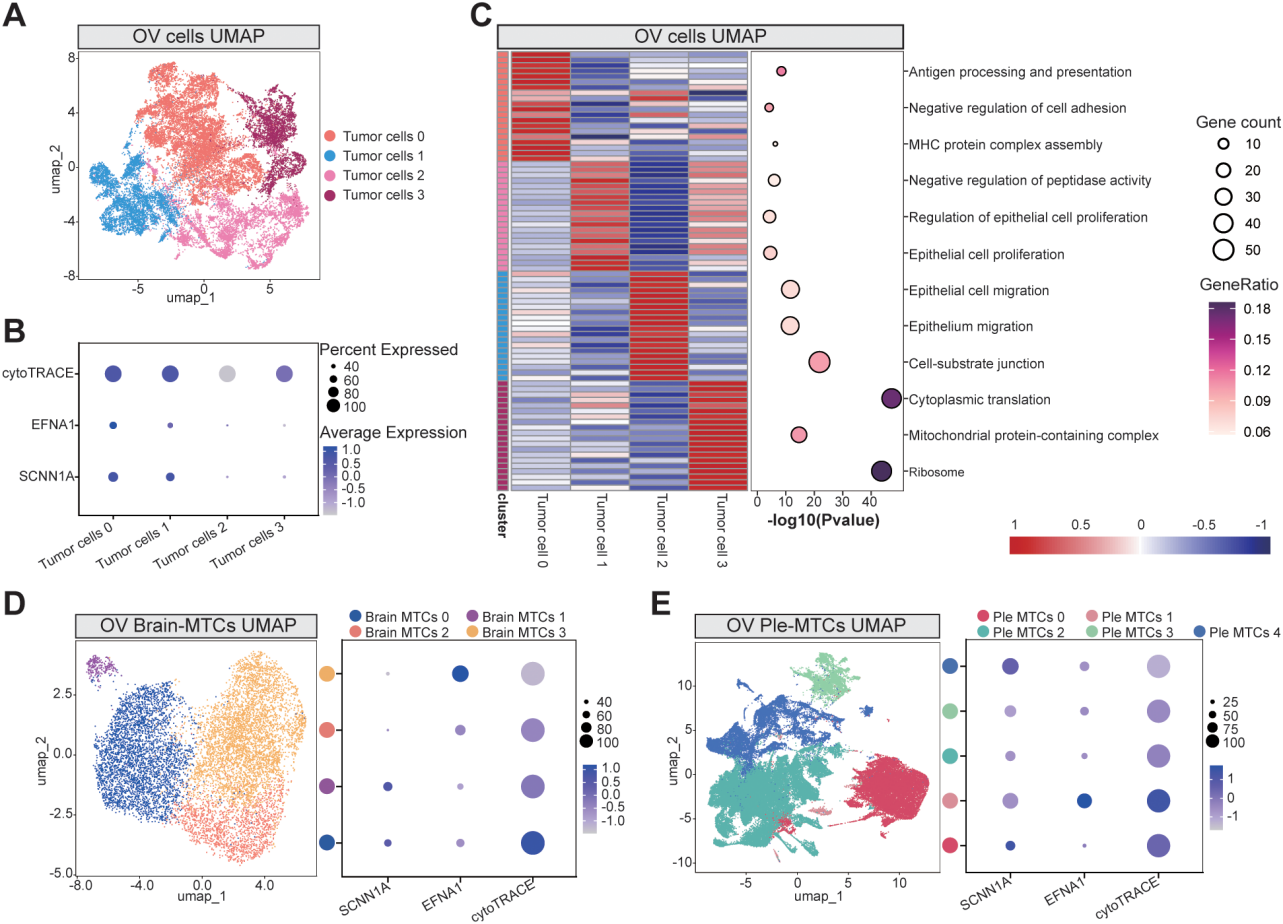
Tumor cell subcluster analysis: **(A)** UMAP visualization of primary OV cell subclusters. **(B)** Bubble plot demonstrating cytoTRACE-predicted differentiation potential across tumor subclusters, with the expression profiles of key genes EFNA1 and SCNN1A. **(C)** GO functional enrichment analysis across the tumor subclusters. **(D, E)** UMAP projections of brain metastasis-derived **(D)** and pleural effusion-associated **(E)** subclusters, paired with bubble plots indicating cytoTRACE scores and marker gene expression levels. Abbreviations: Brain metastasis-derived MTCs cluster: Brain MTCs, Pleural effusion-derived MTCs cluster: Ple MTCs.

We comprehensively analyzed OV cell subclusters to better understand the biological mechanisms by which the key genes SCNN1A and EFNA1 promote OV progression. Tumor cells isolated from OV tissues were reclustered into four distinct subpopulations (**Figure 5**), and their differentiation potentials were assessed using *cytoTRACE*. Bubble plots of EFNA1 and SCNN1A expression levels demonstrated that tumor cells 0 and 1, which exhibited higher differentiation potential, had higher expression of these genes (**Figure 5**). Functional enrichment analysis of the subclusters (**Figure 5**, **Supplementary Table 13**) indicated that cells with higher differentiation potential were significantly enriched in immune-related pathways (antigen processing and presentation and MHC protein complex assembly) and invasion-related pathways (epithelial cell proliferation). Conversely, subclusters with lower differentiation potential were primarily associated with pathways implicated in advanced metastatic processes, including epithelial migration and cell-substrate junction formation. These results indicate that tumor subpopulations with higher EFNA1 and SCNN1A expression may drive metastasis by promoting TME remodeling and initiating tumor proliferation.

We expanded our analysis to MTCs from brain and pleural effusion-derived metastases to further explore these dynamics. Reclustering and cytoTRACE-based assessments of pleural effusion-derived metastases again revealed a positive correlation between the expression of EFNA1 and SCNN1A and the differentiation potential (**Figures 5D**). However, in brain metastases, this correlation was less pronounced, suggesting that the influence of EFNA1 and SCNN1A on differentiation-related pathways may depend on the context. These findings underscore the potential importance of these genes in the early stages of tumor dissemination and their variable roles in distinct metastatic microenvironments.

### 3.5 Cell-cell communication in TME

**Figure 6:**
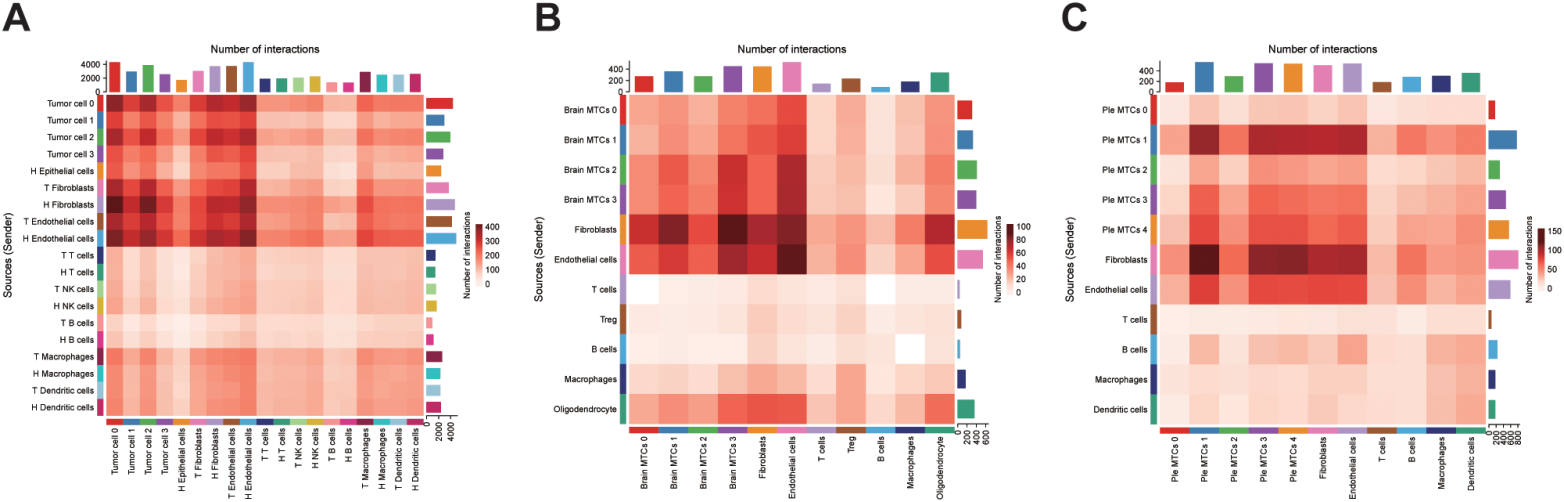
Cell-cell interaction analysis: **(A)** Heatmap of cellular interactions in primary OV samples. **(B, C)** Interaction heatmaps for brain and pleural effusion metastasis samples. Brain metastasis-derived MTCs cluster: Brain MTCs, Pleural effusion-derived MTCs cluster: Ple MTCs.

We performed cell-cell communication analysis in the three sample groups to examine how tumor cell subclusters influence the TME. Using CellChat, we observed that tumor cells interacted extensively with stromal components, particularly fibroblasts and endothelial cells. In primary tumors, tumor cell 0, which displayed a higher differentiation potential, exhibited the strongest interaction with the surrounding cells (**Figure 6**). Brain metastasis-derived MTCs cluster 0 had a higher differentiation potential in brain metastases but exhibited fewer interactions with neighboring cells, indicating a more isolated cellular environment (**Figure 6**). Pleural effusion-derived MTCs cluster 1, which also demonstrated a higher differentiation potential, frequently interacted with other cell types in pleural effusion metastases (**Figure 6**). Pathway-level dissection revealed that tumor cells, particularly those with pronounced stemness, predominantly signal to fibroblasts via the midkine (MK) axis (**Supplementary Figures 3**-**6**). Specifically, high expression of MDK in tumor cells likely engages SDC2 and NCL, both abundantly expressed on fibroblasts, to mediate these interactions (**Supplementary Figure 7**). We propose that this MDK-SDC2/NCL signaling circuit represents a key mechanism by which OV cells remodel their microenvironment and promote metastatic dissemination (26).

## 4 Discussion

OV is a highly aggressive gynecologic malignancy with a poor prognosis, largely due to late-stage diagnosis and a propensity for metastasis (27). Although advances in diagnostic and therapeutic modalities have been achieved in recent years, the limitations of current biomarkers, combined with the complexity of the TME and intrinsic heterogeneity of OV, pose significant challenges for identifying novel biomarkers and elucidating metastatic mechanisms (28). The emergence of multi-omics technologies, particularly scRNA-seq and exosome profiling, has created new opportunities to address these challenges. scRNA-seq enables high-resolution transcriptomic analysis at the single-cell level, allowing for the precise identification of various cellular subpopulations within tumors and their microenvironments. This technique also reveals the distinct functional roles and signaling pathways in tumorigenesis, metastasis, and drug resistance, providing a theoretical basis for precision medicine (19). Concurrently, exosomes serve as pivotal mediators of intercellular communication by transferring bioactive molecules, such as mRNA, microRNA, and proteins, effectively regulating recipient cell behavior and promoting tumor growth, metastasis, and immune evasion (29, 30). Together, these approaches deepen our understanding of OV metastasis and present innovative avenues for biomarker identification.

This study integrated scRNA-seq, bulk RNA-seq, and circulating exosomal RNA-seq data to systematically analyze the correlation between OV heterogeneity and exosomal RNA expression profiles. Our findings demonstrated that epithelial cell populations exhibited the most pronounced gene expression variations, underscoring their critical involvement in tumor biology. Consistent with these findings, Xu et al. demonstrated that the high heterogeneity of tumor epithelial cells in breast cancer is closely associated with specific gene expression patterns, enabling their subdivision into subpopulations linked to lymph node metastasis and therapeutic targets (31). Moreover, we identified 52 common genes in primary tumor epithelial cells, MTCs, and exosomes. Subsequent qPCR and IHC findings confirmed that SCNN1A and EFNA1 were highly expressed in both *in situ* carcinoma and OV-derived exosomes, supporting their potential as reliable biomarkers for OV. Based on the expression levels of these key genes, we developed a diagnostic model that effectively distinguished OV cases via blood-based exosomal gene detection, with an impressive AUC of 0.948. Wang et al. performed large-scale studies to validate the utility of exosome-based biomarker models for early tumor diagnosis (32). Our non-invasive detection system demonstrates promise for the early identification of high-risk metastatic cases, potentially facilitating precision intervention strategies and improving patient survival.

Furthermore, our findings suggest that tumors may remodel their microenvironment by releasing exosomes, thereby establishing niches conducive to metastasis. Detailed analyses of metastatic samples from patients with brain metastases and malignant pleural effusions revealed the dynamic evolution of important cellular subtypes during metastasis. Tumor cells exhibited significant enrichment of exosome-associated genes, most notably SCNN1A and EFNA1, which were significantly upregulated in metastatic lesions and correlated with a poorer prognosis. IHC analysis demonstrated a progressive increase in SCNN1A and EFNA1 expression from adjacent non-tumor tissue to primary tumors and metastatic foci, with notably higher levels in subpopulations of tumor cells exhibiting a higher differentiation capacity. These observations imply that these genes may be important for maintaining stem cell-like characteristics, adapting to new microenvironments, and promoting efficient metastasis. Functional pathway analyses further revealed that these cellular subpopulations were significantly enriched in immune-related pathways, suggesting that SCNN1A and EFNA1 may contribute to immune evasion and the development of a metastasis-supportive TME. Furthermore, Morrissey et al. reported that exosomes secreted by primary tumors can induce PD-L1 upregulation and increase lactate secretion in tissue-resident macrophages in the pre-metastatic niche, thereby fostering an immunosuppressive environment that promotes tumor spread (33). Notably, in brain metastatic samples, the highly differentiated “brain MTC0–1 cluster” did not indicate the expected increase in intercellular interactions, which may be attributed to the restrictive nature of the blood-brain barrier and the brain’s immune-privileged status that allows tumor cells to evade immune surveillance without extensive cell-to-cell communication (34–36).

Recent studies have elucidated the biological roles of SCNN1A and EFNA1 in cancer. SCNN1A, which encodes the α-subunit of the epithelial sodium channel, is aberrantly expressed in OV, pancreatic, and other cancers. It is associated with increased tumor cell proliferation, migration, and invasiveness (37, 38). As a key ion channel component, SCNN1A regulates intracellular and extracellular sodium balance to maintain cellular homeostasis, adhesion, and migration. Its dysregulation may promote epithelial–mesenchymal transition, which promotes metastasis (39). Furthermore, SCNN1A-mediated sodium transport may alter extracellular matrix composition and intercellular interactions, further exacerbating tumor aggressiveness and the risk of distant metastasis (40). EFNA1, a cell surface ligand, primarily interacts with the EphA2 receptor to regulate tumor cell migration, invasion, and proliferation, and its overexpression is closely associated with tumor progression in numerous cancers (41). For instance, Jiao et al. demonstrated that EFNA1 activation of EphA2 causes cytoskeletal remodeling and downstream signaling, promoting tumor cell migration and invasion (42). Similarly, Chu et al. reported that higher EFNA1 expression is linked to increased invasiveness and metastatic potential in gastric cancer, indicating its viability as an early biomarker (43). Shiuan et al. demonstrated that the loss of host EFNA1 significantly reduced the metastatic risk in a murine breast cancer model (44). Furthermore, pan-cancer analyses have indicated that EFNA1 and its family members are closely associated with immune cell infiltration in the TME, highlighting their potential role in immune evasion (45). Collectively, these studies reinforce the pivotal roles of SCNN1A and EFNA1 in OV progression and metastasis, supporting their potential as biomarkers.

Despite these promising findings, our study has several limitations. First, the relatively small sample size necessitates the validation of the diagnostic utility of these key biomarkers in larger cohorts. Second, the mechanistic pathways underlying the transfer of these molecules from intracellular compartments to circulating exosomes have not been thoroughly elucidated. Finally, comprehensive multi-omics investigations, such as transcriptomics, proteomics and metabolomics, are required to delineate the precise biological functions of these biomarkers in tumorigenesis and disease progression.

In conclusion, by integrating single-cell sequencing with exosomal RNA profiling, we systematically delineated the heterogeneity and metastatic mechanisms of OV and successfully identified SCNN1A and EFNA1 as key exosomal biomarkers. Based on these markers, we developed an RF diagnostic model that demonstrated high accuracy in early OV detection and metastatic risk prediction, providing robust support for clinical precision diagnostics. Moreover, functional assays confirmed that SCNN1A and EFNA1 are highly expressed in well-differentiated tumor cell subpopulations and contribute to tumor progression by modulating cell migration, invasion, and immune evasion. Importantly, these biomarkers also play important roles in metastatic TME, potentially facilitating tumor adaptation to distant sites via exosome-mediated intercellular communication.

## Data Availability

The datasets presented in this study can be found in online repositories. The names of the repository/repositories and accession number(s) can be found in the article/**Supplementary Material**.

## Supplementary material

Supplementary Figure 1 Exosome validation: **(A)** Transmission electron microscopy image reveals the characteristic spherical vesicular structures of exosomes isolated from plasma, confirming their identity. **(B)** Western blot validation of exosome markers (CD63, CD81, and TSG101) confirming the presence and quality of the isolated exosomes.

Supplementary Figure 2 SLC39A4 clinical correlation analysis.

Supplementary Figure 3 CellChat cell-cell interaction map.

Supplementary Figure 4 CellChat *in situ* cancer cell interaction pathways heatmap.

Supplementary Figure 5 CellChat analysis of OV pleural effusion metastasis cell interaction pathways heatmap.

Supplementary Figure 6 CellChat analysis of OV brain metastasis cell interaction pathways heatmap.

Supplementary Figure 7 Bubble plots of CellChat-inferred ligand–receptor interactions in the midkine (MK) signaling pathway between tumor cells and fibroblasts: **(A)** Interactions in primary OV. **(B)** Interactions in OV brain metastases. **(C)** Interactions in OV pleural effusion metastases.

Supplementary Table 1 Results of exosome nanoparticle-tracking analysis.

Supplementary Table 2 qPCR primer table.

Supplementary Table 3 Differential analysis results of TCGA OV data.

Supplementary Table 4 Differential analysis results of blood-derived exosome OV data.

Supplementary Table 5 Differential analysis results of scRNA-seq data from OV tissue.

Supplementary Table 6 Differential analysis results of scRNA-seq data from OV brain metastases.

Supplementary Table 7 Differential analysis results of scRNA-seq data from pleural effusion OV metastasis.

Supplementary Table 8 GO functional enrichment analysis of high-expression genes in OV brain-metastasis cell-specific genes.

Supplementary Table 9 GO Functional enrichment analysis of high-expression genes in OV pleural effusion metastasis cell-specific genes.

Supplementary Table 10 Differential gene intersection results.

Supplementary Table 11 Survival and clinical correlation analysis results for the key genes.

Supplementary Table 12 Diagnostic performance metrics of the different classification models.

Supplementary Table 13 GO functional enrichment analysis of highly expressed genes in OV cell subclusters.
