# Supplementary figures and images for: Integrative single-cell and exosomal multi-omics uncovers SCNN1A and EFNA1 as non-invasive biomarkers and drivers of ovarian cancer metastasis
Source: Front Immunol. 2025 Jul 25;16:1630794. doi: 10.3389/fimmu.2025.1630794 (PMC12331593; doi:10.3389/fimmu.2025.1630794)

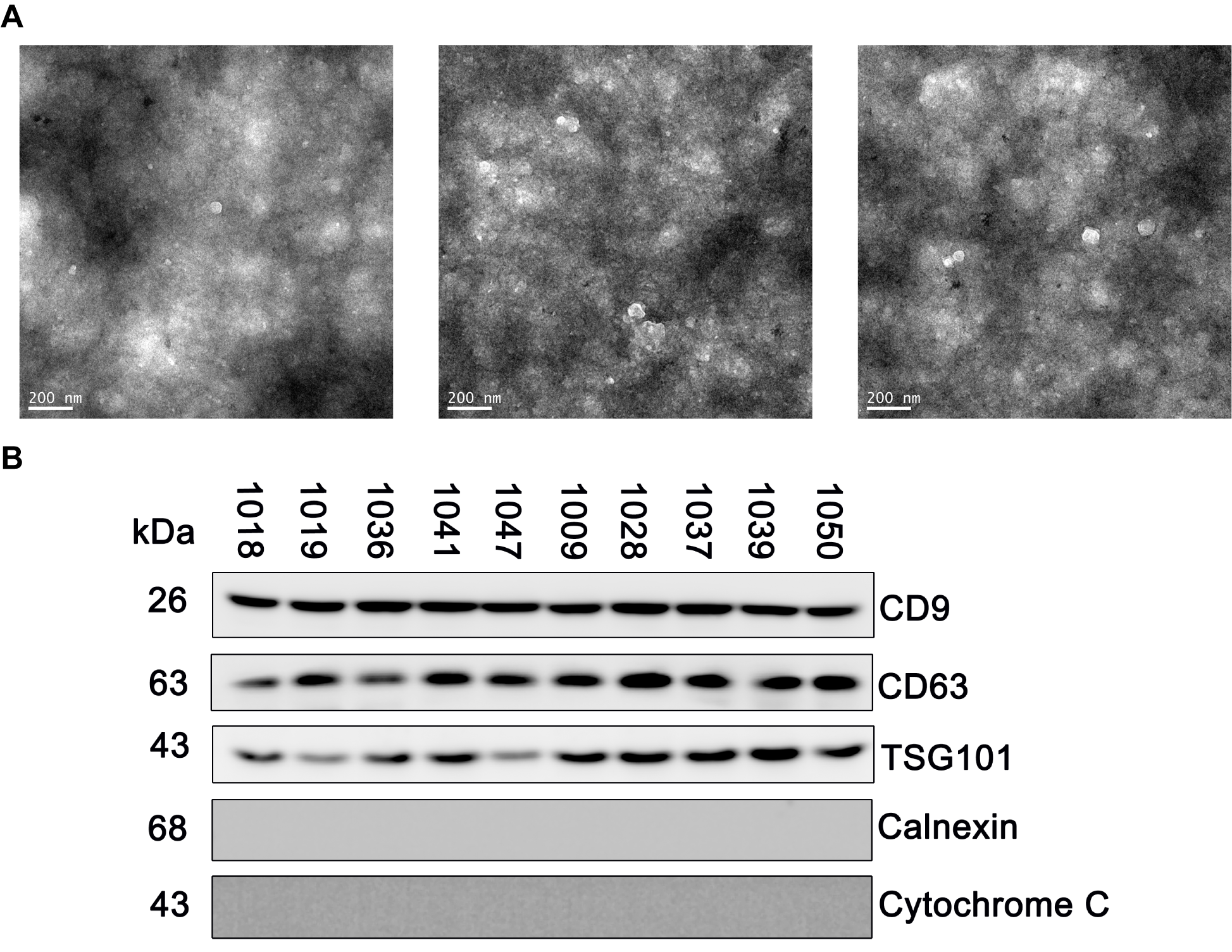

Supplement: Supplementary Figure 1 — Exosome validation: (A) Transmission electron microscopy image reveals the characteristic spherical vesicular structures of exosomes isolated from plasma, confirming their identity. (B) Western blot validation of exosome markers (CD63, CD81, and TSG101) confirming the presence and quality of the isolated exosomes. [file Image1.tif]

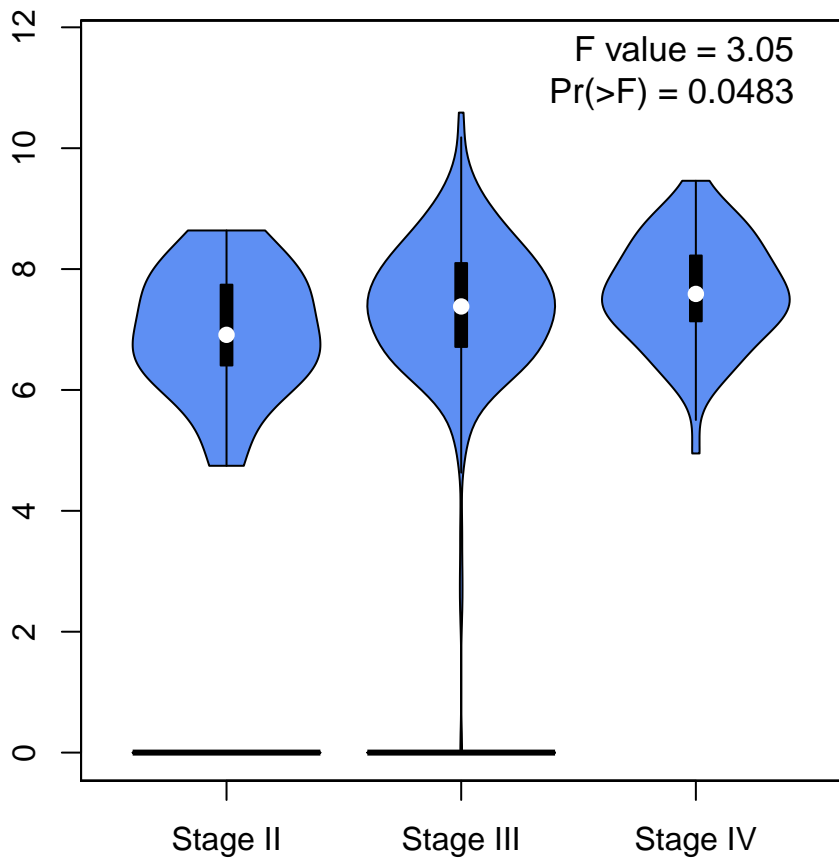

Supplement: Supplementary Figure 2 — SLC39A4 clinical correlation analysis. [file Image2.pdf]

## OV tumor cells

Number of interactions

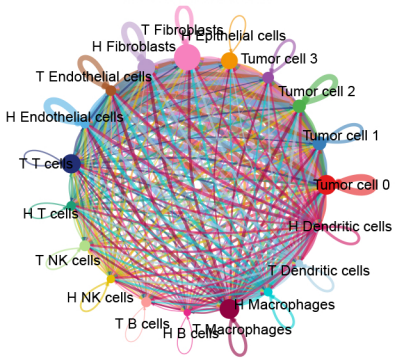

## OV ple-MTCs

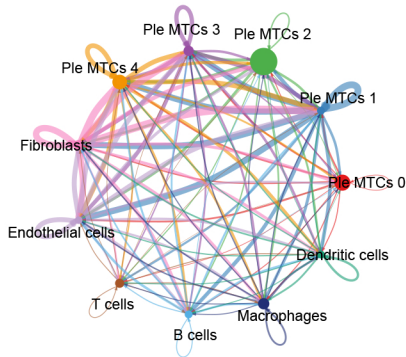

## OV brain-MTCs

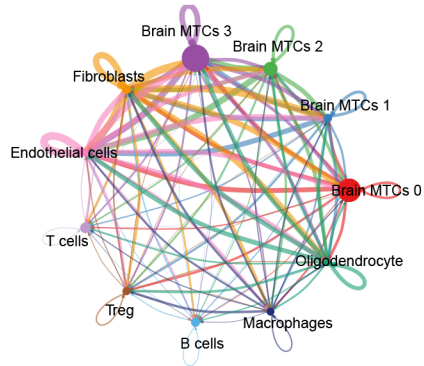

Supplement: Supplementary Figure 3 — CellChat cell-cell interaction map. [file Image3.pdf]

Overall signaling patterns

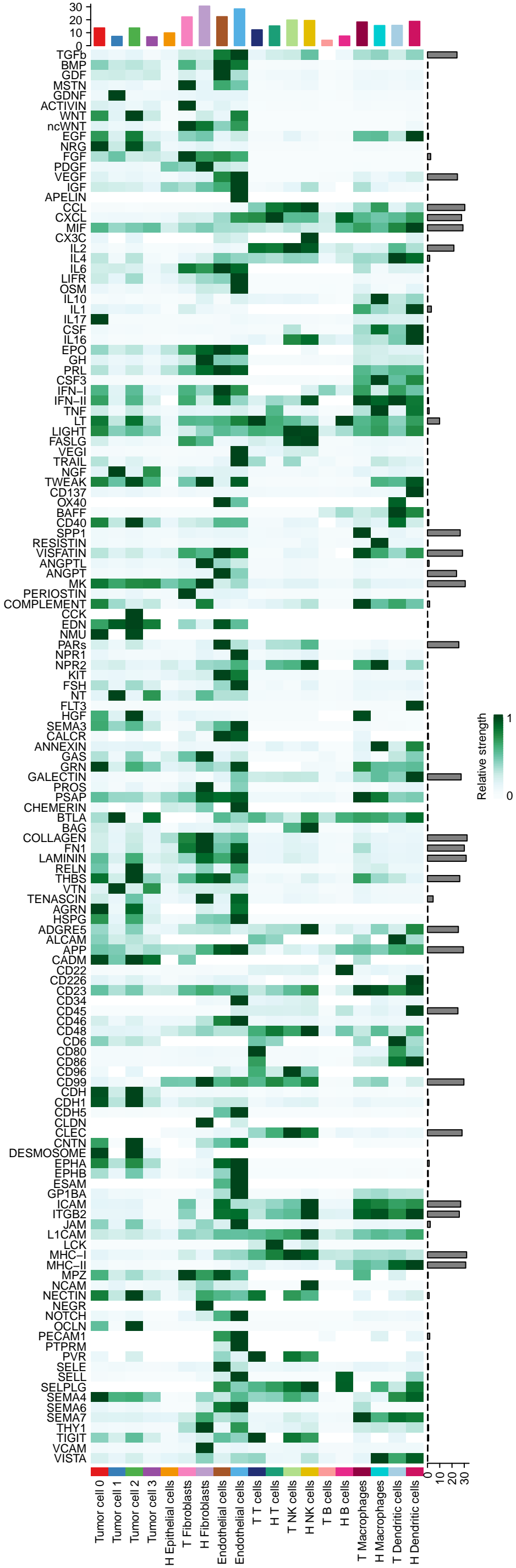

Supplement: Supplementary Figure 4 — CellChat in situ cancer cell interaction pathways heatmap. [file Image4.pdf]

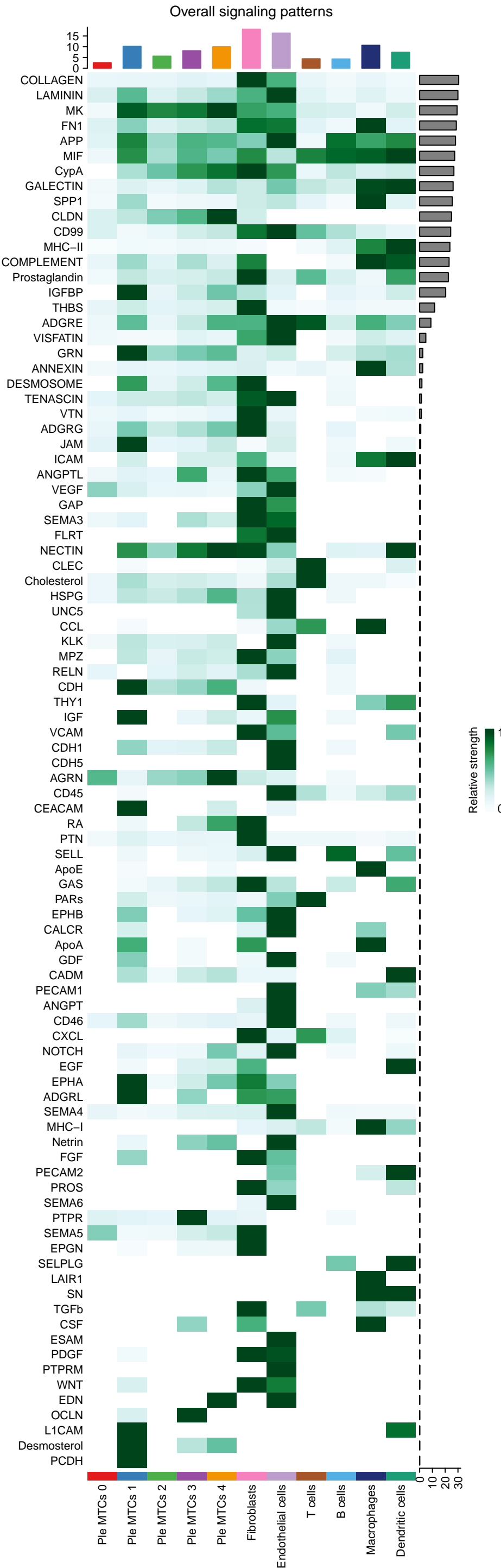

Supplement: Supplementary Figure 5 — CellChat analysis of OV pleural effusion metastasis cell interaction pathways heatmap. [file Image5.pdf]

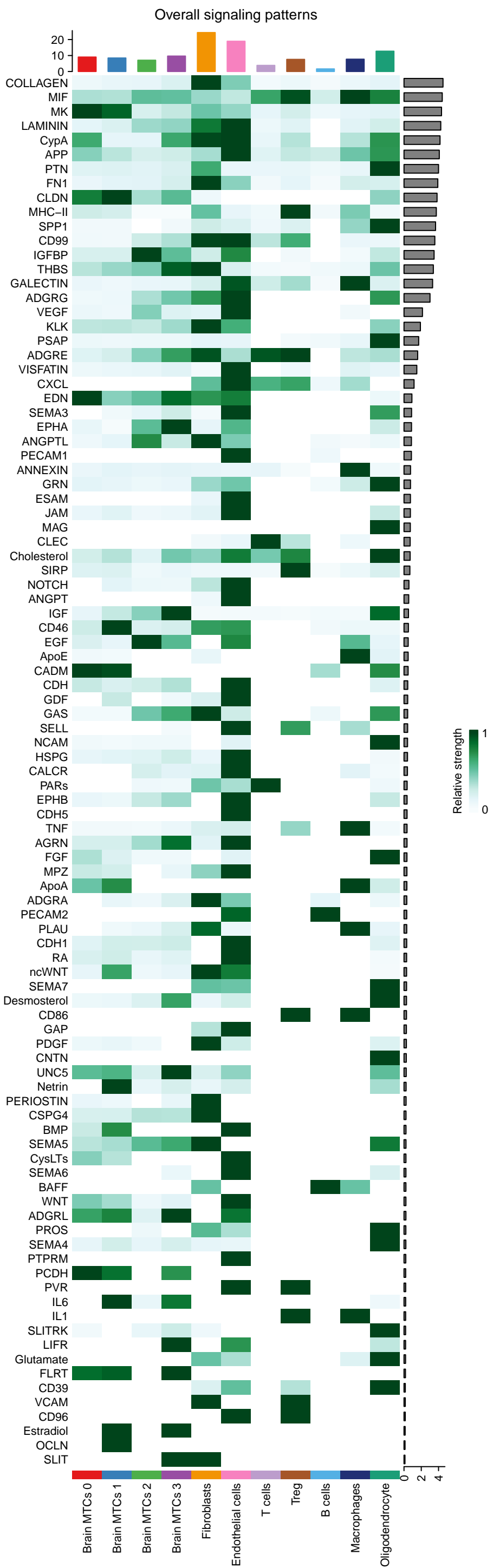

Supplement: Supplementary Figure 6 — CellChat analysis of OV brain metastasis cell interaction pathways heatmap. [file Image6.pdf]

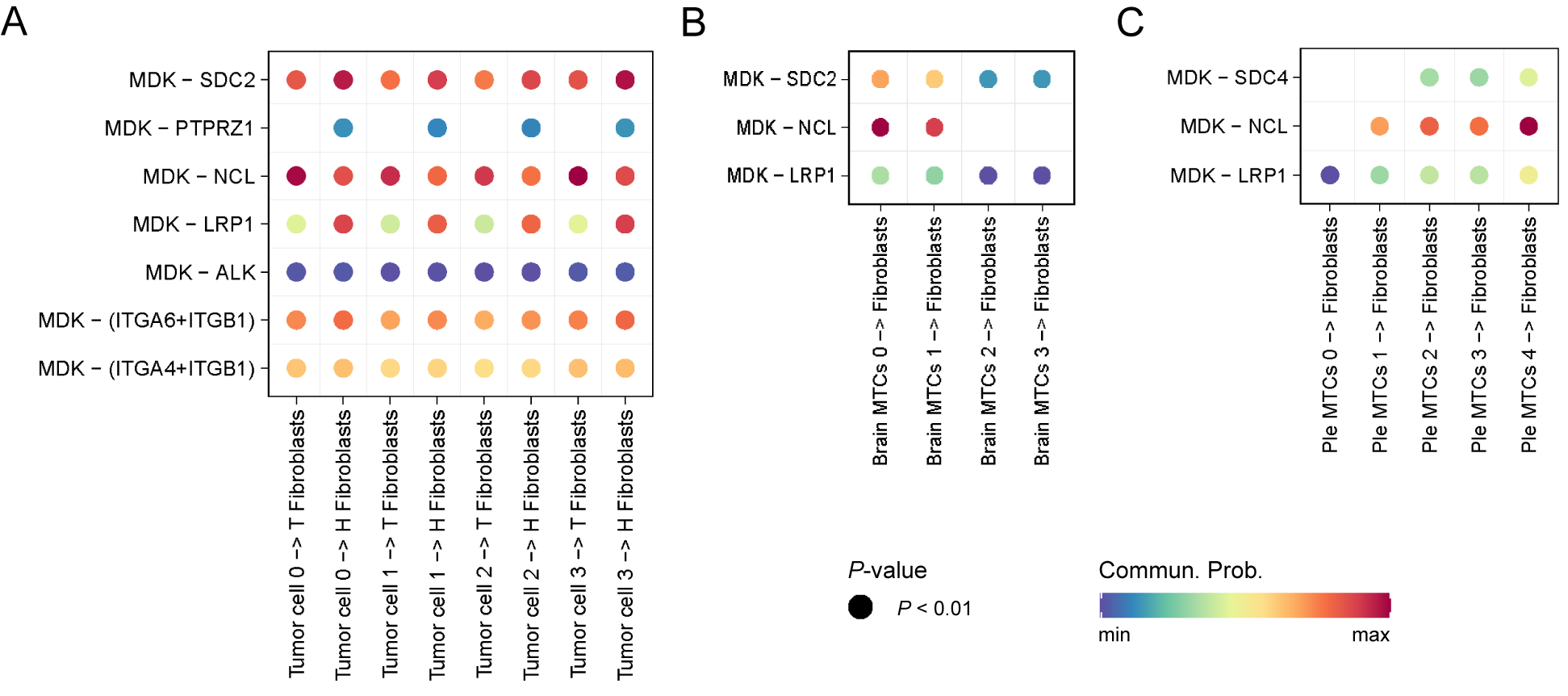

Supplement: Supplementary Figure 7 — Bubble plots of CellChat-inferred ligand–receptor interactions in the midkine (MK) signaling pathway between tumor cells and fibroblasts: (A) Interactions in primary OV. (B) Interactions in OV brain metastases. (C) Interactions in OV pleural effusion metastases. [file Image7.tif]
